# Functionally Active Antibodies to the Angiotensin II Type 1-Receptor Measured by a Luminometric Bioassay Do Not Correlate With Clinical Manifestations in Systemic Sclerosis: A Comparison With Antibodies to Vascular Receptors and Topoisomerase I Detected by ELISA

**DOI:** 10.3389/fimmu.2021.786039

**Published:** 2021-12-09

**Authors:** Lukas Bankamp, Beate Preuß, Ann-Christin Pecher, Nicola Beucke, Jörg Henes, Reinhild Klein

**Affiliations:** Department of Internal Medicine II, University of Tuebingen, Tuebingen, Germany

**Keywords:** systemic sclerosis, functionally active autoantibodies, angiotensin II type-1 (AT1) receptor, luminometric assay, anti-topoisomerase I antibody (Scl70)

## Abstract

**Objectives:**

1) To detect functionally active antibodies(abs) to the angiotensin II type-1-receptor (AT_1_R) by a novel luminometric assay. 2) To assess their prevalence in systemic sclerosis (SSc), other collagen disorders, as well as in further chronic inflammatory disorders including autoimmune, toxic and chronic viral diseases. 3) To compare these abs with anti-AT1R antibodies by ELISA as well as with antibodies to endothelin-type-A receptors (ET_A_1) and to topoisomerase I (topo-I) with respect to their specificity and clinical relevance.

**Methods:**

Sera from 98 SSc-patients, 110 patients with other chronic inflammatory rheumatic disorders, 97 patients with autoimmune liver diseases, 57 patients with toxic or chronic viral liver diseases and 36 healthy controls were analyzed. A luminometric bioassay was established with Huh-7-cells constitutively expressing the AT_1_R. Patients’ sera were also tested by commercially available ELISA for anti-AT_1_R, -ET_A_1- and by an in-house ELISA for anti–topo-I-abs.

**Results:**

Fifty-two percent of the SSc-patients had functionally active anti-AT_1_R-abs with stimulatory (34%) or inhibitory capacity (18%). They were present also in up to 59% of patients with other rheumatic diseases but only 22% of healthy individuals (sensitivity 52%, specificity 53%). The functionally active antibodies detected by the luminometric assay did not correlate with anti-AT_1_R-, -ET_A_1- or -topo-I-abs measured by ELISA, but there was a strong correlation between anti-topo-I-, AT_1_R-, and -ET_A_1-ab reactivity measured by ELISA. Sensitivities of 55%, 28% and 47% and specificities of 66%, 87%, and 99% were calculated for these anti-AT_1_R-, -ET_A_1-, and anti-topo-I-abs, respectively. Functionally active abs did not correlate with disease severity or any organ manifestation. In contrast, abs to topo-I, AT_1_R, and ET_A_1 were associated with digital ulcers, pulmonary- and esophageal manifestation.

**Conclusions:**

Functionally active anti-AT_1_R-abs can be detected in SSc-patients but do not correlate with disease activity. They are not specific for this disease and occur also in other autoimmune disorders and even viral or toxic diseases. Also, the vascular antibodies detected by ELISA are not SSc-specific but correlated with disease manifestations. In contrast, anti-topo-I-abs were confirmed to be a highly specific biomarker for both, diagnosis and organ manifestations of SSc.

## Introduction

Antinuclear antibodies reacting for instance with topoisomerase-I (anti-topo-I; formerly known as anti-Scl70), centromeres (ACA) or some nucleolar antigens (i.e. fibrillarin) are a hallmark of systemic sclerosis (SSc) (1). Anti-topo-I antibodies are more prevalent in – but not restricted to - diffuse cutaneous (dc) SSc whereas ACA are more frequent in limited cutaneous (lc) SSc (2–4). They are useful markers for diagnosis and prognosis of organ involvement but their contribution to disease pathogenesis is still under investigation (2, 5, 6).

In some organ specific autoimmune diseases such as Graves’ disease, myasthenia gravis or idiopathic cardiomyopathy (7–9) also functionally active antibodies have been observed inhibiting or stimulating receptors on cell membranes; they may be potentially pathogenic and responsible for different clinical manifestations. Meanwhile they have been found also in systemic autoimmune disorders as for instance autoantibodies to the muscarinic acetylcholine receptors of the M3-type in primary Sjoegren syndrome (pSS) (10, 11) or to angiotensinII-type1- and endothelin-type-A-receptors (AT_1_R, ET_A_1) in SSc (12). The first studies on anti-AT_1_R- and -ET_A_1-antibodies were based on true functional assays, namely bioassays that involved spontaneously beating cultured rat cardiomyocytes (13, 14) or human endothelial cells (14, 15). Furthermore, a functional assay measuring AT_1_R-like autoantibody reactivity with AT_1_R-transfected Chinese hamster ovary (CHO)-cells using β-arrestin activation as the parameter has been described (16, 17). The antibodies have been hypothesized to play a pathogenetic role in SSc although they are not specific for the disease and have been also observed for instance in malignant hypertension, primary aldosteronism, pregnant women with pre-eclampsia, Alzheimer’s disease, and renal or heart graft failure after transplantation (13, 14, 17–20). Since those bioassays are time consuming and difficult to standardize for routine use, solid phase assays were established with extracts from CHO-cells overexpressing the human AT_1_R or ET_A_1 (15). They are meanwhile commercially available and have been applied in several studies analyzing the clinical relevance of these antibodies in SSc. Patients with high anti-AT_1_R or ET_A_1-antibodies have been shown to have a high risk for diffuse SSc and complications such as pulmonary hypertension, lung fibrosis and digital ulcers and also predicted disease related mortality (21, 22). However, they have been found also in other disorders such as renal allograft-reaction, hypertension, primary aldosteronism, Alzheimer’s disease, chronic graft versus host disease after stem cell transplantation and even COVID-19 infection (14, 16–19, 23–26).

Aim of the present study was, therefore, to determine the occurrence of functionally active anti-AT_1_R antibodies in SSc and other connective tissue disorders by a newly developed luminometric assay using Chinese hamster ovary (CHO-K1) cells transfected with the AT_1_R plasmid DNA thus overexpressing the receptor but also a human cell line, Huh7, known to constitutively express AT_1_R. This assay was compared with commercially available ELISA for the detection of anti-AT_1_R antibodies but also with the presence of anti-topo-I antibodies. Furthermore, we re-analyzed the relevance of these different antibodies with respect to clinical manifestations.

Moreover, we wanted to see whether these antibodies also occur in patients with other well-defined autoimmune disorders or in those with toxic and viral disorders in order to get more insights into their mode of induction as well as their pathophysiological role.

## Patients

Sera from 98 SSc-patients (87 females, 11 males) were analyzed. Clinical details of these patients are given in the supplement (**Supplement Table S1**). In all patients, diagnosis was in accordance to the 2013 Classification Criteria for SSc (27). Seventy-one suffered from lcSSc and 27 from dcSSc. All patients were assessed for different organ manifestations. High resolution computer tomography (HRCT) scan was performed for pulmonary assessment, and echocardiography, NT-proBNP and troponin-I for monitoring cardiac damage as recently described (28). Thirty-one patients had antibodies to Scl70 in the immunodiffusion, 25 antibodies to nucleoli (fibrillarin) and 31 antibodies to centromeres (ACA) in the immunofluorescence test.

As controls, sera from 70 patients with other connective tissue diseases were included: systemic lupus erythematosus [SLE] being positive for antibodies to double stranded DNA, SSA/Ro or Sm: n=21; mixed connective tissue disease [MCTD] being anti-RNP positive: n=25, primary Sjoegren syndrome [pSS] positive for anti-SSA/Ro: n=24. Furthermore, sera from 24 patients with rheumatoid arthritis (RA) being anti-CCP positive, and 16 patients with polymyalgia rheumatica (PM) being antibody negative were included. All patients with rheumatic diseases were seen by one of the authors (JH, ACP); diagnosis had been established according to international criteria.

In order to see whether those antibodies may occur also in patients with other well defined autoimmune disorders we included patients with autoimmune liver disorders, i.e. primary biliary cholangitis (PBC, n=37, all positive for antibodies to antimitochondrial antibodies reacting with the 2-oxo-dehydrogenas complex), autoimmune hepatitis (n=30, all positive for antibodies to nuclei and smooth muscle antigens/actin) and 30 patients with primary sclerosing cholangitis (all positive for antibodies to neutrophils, pANCA).

Sera from 36 healthy individuals (students, technical staff) were included as controls.

Age and sex distribution of all patients are given in the supplement (**Supplement Table S2**).

The study had been approved by the local ethical committee (No. 076/212BO1; 647/2016BO2); it was performed according to the Helsinki guidelines, and patients had given written informed consent before the study.

## Methods

### Purification of Immunoglobulins From Patients’ Sera

For the functional assay immunoglobulins were isolated from patients’ sera in order to avoid a non-specific effect of other components in the sera on AT_1_R-activity. They were isolated from patients’ sera by ammonium sulphate precipitation previously shown to give pure immunoglobulin fractions and more reliable results than immunoglobulins purified by other methods (11). To 300µl serum the equal amount of a saturated ammonium sulphate solution (76.7g/100ml H_2_O) was slowly added. After precipitation overnight at 4°C the sample was centrifuged at 5,000 g for 30 min. The supernatant was discarded and the precipitate was washed twice with a 60% ammonium sulphate solution and centrifuged at 5,000 g for 15 min. Finally, the purified immunoglobulins were dissolved in 300µl with Hank’s balanced salt solution (HBSS). Protein concentrations were about 10µg/µl.

All samples were stored at -20°C.

### Preparation of Plasma Membranes and Analysis by Western Blotting

Plasma membranes were prepared from Chinese hamster ovarian cells (CHO-K1) overexpressing the AT_1_R and the human cell line Huh7 expressing constitutively the AT_1_R (29) according to standardized methods (30, 31). They were then applied to Western blotting (WB) for the analysis of receptor expression and purity (**Supplement Figure S1**).

The plasma membranes were analysed by sodium dodecyl sulphate-polyacrylamide gel electrophoresis (SDS-PAGE) using a 4.5% stacking and a 10% running gel according to a standard procedure. 10 µg protein were applied to each lane. After transfer of the proteins to nitrocellulose membranes (Li-Cor Inc., USA) the membranes were blocked with 3% bovine serum albumin (BSA) in phosphate buffered saline (PBS) for 60 minutes and incubated with an anti-AT_1_R antibody from rabbit (Biozol, Eching, Germany) at 4°C overnight. The sheets were then incubated with peroxidase conjugated swine anti-rabbit antibody (DAKO, Hamburg, Germany) for 1 hour at room temperature and visualized using 3-amino-9-ethyl carbazole as substrate.

For the analysis of the purity of the membrane fractions, the sheets were also tested against sera from a patient with primary biliary cholangitis (PBC) showing anti-M2-antibodies reacting with the pyruvate dehydrogenase complex (PDC), a patient with systemic sclerosis reacting with topoisomerase I, a patient with mixed connective tissue disease recognizing snRNP68, and a healthy blood donor.

### Methods for Detection of Autoantibodies

#### Immunodiffusion

Antibodies to Scl70 were determined by radial immunodiffusion using extractable nuclear antigens from calf thymus as described previously (32). Anti-Scl70-positive marker sera had been primarily provided by the American Centre of Disease Control, Atlanta, and have been substituted in past years with our own marker sera.

#### Immunofluorescence Test (IF)

For the demonstration of antibodies to nucleoli (fibrillarin) and centromeres sera were tested by immunofluorescence test (IFT) using Hep2-cells as substrate (32).

#### Enzyme Linked Immunosorbent Assay (ELISA)

Anti-topo-I antibodies were analyzed by a published in-house enzyme linked immunosorbent assay (ELISA) using a recombinant full-length topoisomerase-I (Diarect, Freiburg, Germany) (32). Positive and negative standard sera were used in each test to calculate a standard curve. Results are given as absorbance x1.000. This assay is routinely performed in our lab since several years giving reliable and reproducible results.

For the demonstration of anti-AT_1_R antibodies to two different ELISA-kits were used: one quantitative ELISA from CellTrend GmbH (Luckenwalde, Germany) and one competitive ELISA kit from MyBioSource (San Diego, CA, USA). They were performed according to the instructions of the companies. Results are given as U/ml (CellTrend) or ng/ml (MyBioSource).

Furthermore, sera were tested for anti-ET_A_1 antibodies by ELISA (CellTrend GmbH).

#### Bioassay Measuring Functionally Active Anti-AT_1_R Antibodies

For this assay, CHO-K1-cells stably transfected with an aequorin/green fluorescence fusion plasmid were transiently transfected with an AT_1_R plasmid DNA analogous to the protocol described for the muscarinic M3 receptor (11) using FuGENE6 reagent (Promega, Madison, WI, USA). Optimal concentrations of cells, AT_1_R plasmid DNA, and FuGENE6 reagent were determined prior to the experiments by serial dilutions. The cells were incubated with 30 µg ammonium sulphate precipitated immunoglobulins from patients’ sera for 1h; then 10 µM of the AT_1_R agonist angiotensin II (Sigma-Aldrich, St. Louis, MI, USA) were added to the cells immediately before the measurement. The change in intracellular [Ca^++^] during 20s was then determined by measuring the emitted light with a 2460 MicroBeta^2^ LumiJET luminometer (Perkin Elmer, Downers Grove, IL, USA). Measurements were performed in quadruplicate.

Moreover, the human cell line Huh7 expressing constitutively the AT_1_R was applied in this assay (29). Cells were transfected with an aequorin/green fluorescence protein fusion plasmid.

As a positive control for the validity of the assay the AT_1_R specific antagonist Losartan (Sigma-Aldrich, St. Louis, MI, USA) was added to the cells in final concentrations ranging from 0.1pM-1µM.

Results were given as absolute RLUs (relative light units) or - when applying serum immunoglobulins - as percentage of RLUs without added immunoglobulins.

### Determination of Normal Values

In the assay measuring functionally active anti-AT_1_R-antibodies, RLU were measured with each immunoglobulin fraction and given as percentage of RLUs (%RLU) without added immunoglobulins; in order to determine a normal range, the immunoglobulins from 36 healthy individuals were analyzed, and the mean of the obtained %RLU was calculated. RLU from patients and controls were divided by this mean resulting in a factor. A factor ≤0.6 was defined as inhibitory activity, a factor ≥1.4 as stimulatory activity.

Cut off values for anti-AT_1_R- and anti-ET_A_1-assays given by the company are defined as 17 U/ml. Re-analyzing them with sera from 15 healthy individuals and calculating the mean of their reactivities +3-fold standard deviation (SD), we found similar values in our settings. Normal values for the anti-topo-I-ELISA had been determined in previous studies with large numbers of healthy individuals. Mean of absorbance (x 1000) +3-fold standard deviation resulted in a cut off > 300. It was re-analyzed in the present study with sera from 15 healthy individuals and found to be identical.

### Statistics

For statistical analysis, SPSS version 15.0 and GraphPad Prism7 were used. Non-parametric tests were applied. Paired data were analyzed by Wilcoxon-, unpaired data by Mann-Whitney U-tests. Fisher’s exact test was used for comparing prevalence. Correlation was evaluated by determination of the Spearman Rank test for non-parametric analyses. Values of p<0.05 were considered statistically significant.

## Results

### Functionally Active Anti-AT_1_R Antibodies Detected by a Luminometric Bioassay

#### Optimization of the Assay

In a first approach we confirmed the expression of AT_1_R by CHO-K1 and Huh7-cells by WB using plasma membranes and an anti-AT_1_R antibody from rabbit (**Supplement Figure S1**).

The luminometric assay was standardized with both cell lines using different cell numbers and FuGENE6 concentrations. Optimal results were obtained with 100.000 cells/ml transfected with 1µg/ml AT_1_R plasmid DNA using a FuGENE6: DNA ratio of 2:1 (data not shown).

The specificity of the assay for the demonstration of anti-AT_1_R antibodies was proven by applying the specific AT_1_R-antagonist Losartan which resulted in an inhibition of AT_1_R activity at 10nM for CHO-K1 and Huh7 cells (**Supplement Figure S2**). This Losartan-induced inhibition was abolished when immunoglobulins with stimulatory antibodies were applied (data not shown).

Since both cell lines gave similar results in the preliminary investigations we preferred for further experiments the human cell line Huh7 constitutively expressing the human AT_1_R.

An intra- and inter-assay coefficient of variation of 20% and 25%, respectively, was calculated.

#### Prevalence and Reactivity of Functionally Active Anti-AT_1_R Antibodies in Patients With SSc and Other Disorders

Immunoglobulins from 73 SSc patients were tested by the luminometric assay. Twenty-five (34%) had antibodies stimulating and 13 (18%) inhibiting receptor activity (total functionally active antibodies: 52%). Similar numbers were observed for patients with other collagen disorders such as pSS and MCTD, and also in PM.

**Figure 1:**
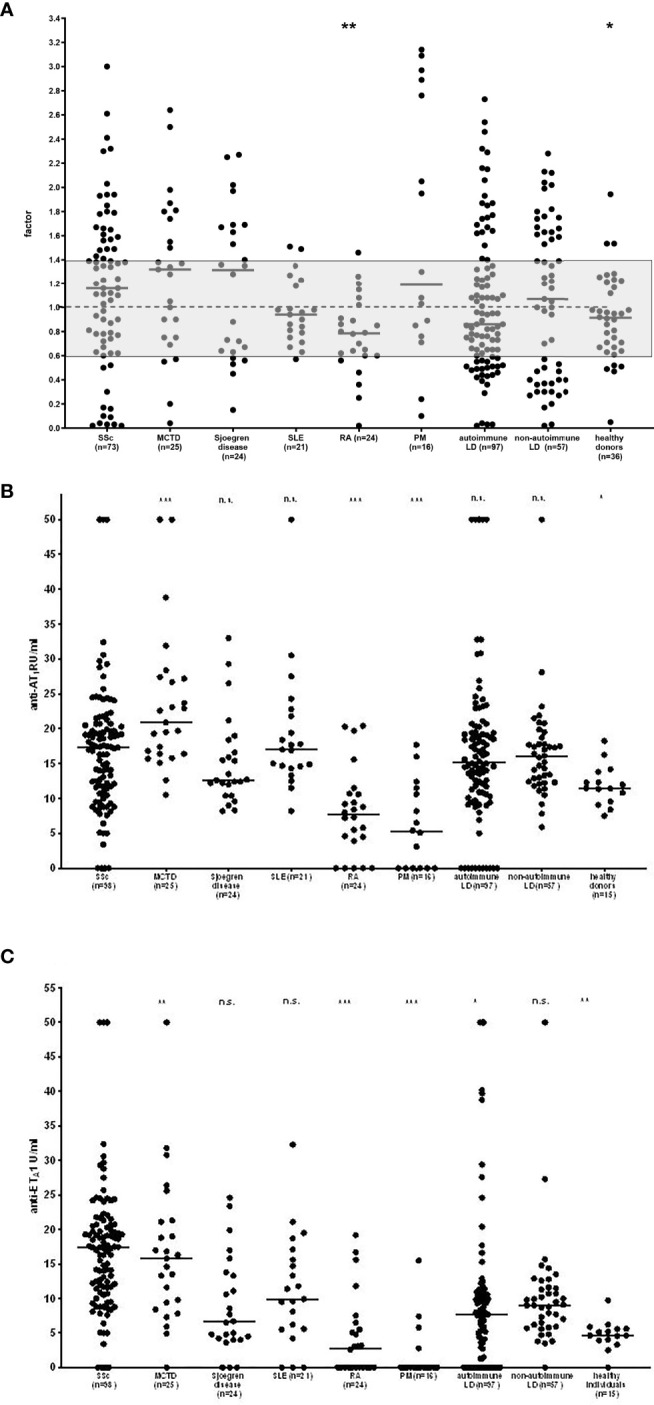
Reactivity of functionally active antibodies to the AT_1_R measured by a luminometric bioassay **(A)** and antibodies to AT_1_R **(B)** and ET_A_1 **(C)** measured by ELISA in patients with systemic sclerosis (SSc) as compared to healthy controls and patients with other collagen disorders as well as further autoimmune and non-autoimmune diseases. For the functional assay, individual values are given reflecting a factor calculated with the mean of healthy individuals. The grey zone indicates the normal range. MCTD-mixed connective tissue disease, SLE-systemic lupus erythematosus, RA-rheumatoid arthritis, PM-polymyalgia rheumatica, LD-liver diseases. p-values for SSc as compared to other disorders are given: n.s., not significant; *p < 0.05; **p < 0.01; ***p < 0.001; **-** median.

In contrast, the prevalence of functionally active and especially stimulatory antibodies was significantly lower in patients with SLE and RA than in SSc (**Table 1** and **Figure 1A**).

**Table 1:** Prevalence of stimulatory and inhibitory antibodies to the AT_1_R in immunoglobulins from patients with different disorders as measured by a luminometric assay.

Diagnosis		Number tested	Antibodies to AT_1_R		
			inhibitory	stimulatory	total
			Number (%) positive		
SSc	total	73	13 (18)	25 (34)	38 (52)
	Anti-Scl70 positive (ID)	24	6 (25)	7(30)	13 (55)
	Anti-Topo I positive (ELISA)	26	5 (19)	5 (19)	10 (38)
	Antibodies to nucleoli (IFT	20	2 (10)	7 (35)	9 (45)
	Antibodies to centromeres (IFT)	25	4 (16)	11 (44)	15 (60)
	No antibodies	4	1 (25)	0	1 (25)
mixed connective tissue disease (MCTD)		25	4 (16)	9 (36)	13 (52)
primary Sjoegren disease (pSS)		24	5 (21)	9 (38)	13 (59)
systemic lupus erythematosus (SLE)		21	1 (5)	2 (10)^*^	3 (15)^*^
rheumatoid arthritis (RA)		24	6 (25)	1 (4)^*^	7 (29)
polymyalgia rheumatica (PM)		16	2 (13)	7 (44)	9 (57)
autoimmune liver diseases (PBC, PSC, AIH)		97	25 (26)	22 (23)	47 (49)
none autoimmune liver diseases (toxic, viral)		57	21 (37)^*^	19 (33)	40 (70)^*^
blood donors		36	5 (14)	3 (8)^*^	8 (22)^*^

Significant as compared to patients with SSc: ^*^p < 0.05.

ID, immunodiffusion; ELISA, enzyme linked immunosorbent assay; IFT, immunofluorescence test.

However, those functionally active anti-AT_1_R antibodies were not only detected in rheumatic disorders but also in other disorders as shown for chronic liver disorders; thus they were present in 49% of patients with autoimmune liver diseases (23% stimulatory, 26% inhibitory) and even 70% of patients with viral or toxic hepatitis (33% stimulatory, 37% inhibitory); especially in patients with non-autoimmune liver diseases the inhibitory antibodies were more frequently found than in SSc patients (and healthy controls) (**Table 1**).

The specificity of this functional assay for SSc was 55%, the sensitivity 52%. Receiver operating curve (ROC)-analysis revealed a significant difference to healthy controls for patients with SSc and MCTD but not for other disorders (**Supplement Table S3**).

Comparing the *reactivities* of the functional antibodies, SSc-patients showed stronger stimulatory properties as compared to patients with RA and healthy donors but not the other groups of patients (**Figure 1A**).

#### Correlation Between the Presence of Functionally Active Anti-AT_1_R Antibodies and Clinical Manifestations in SSc

**Figure 2:**
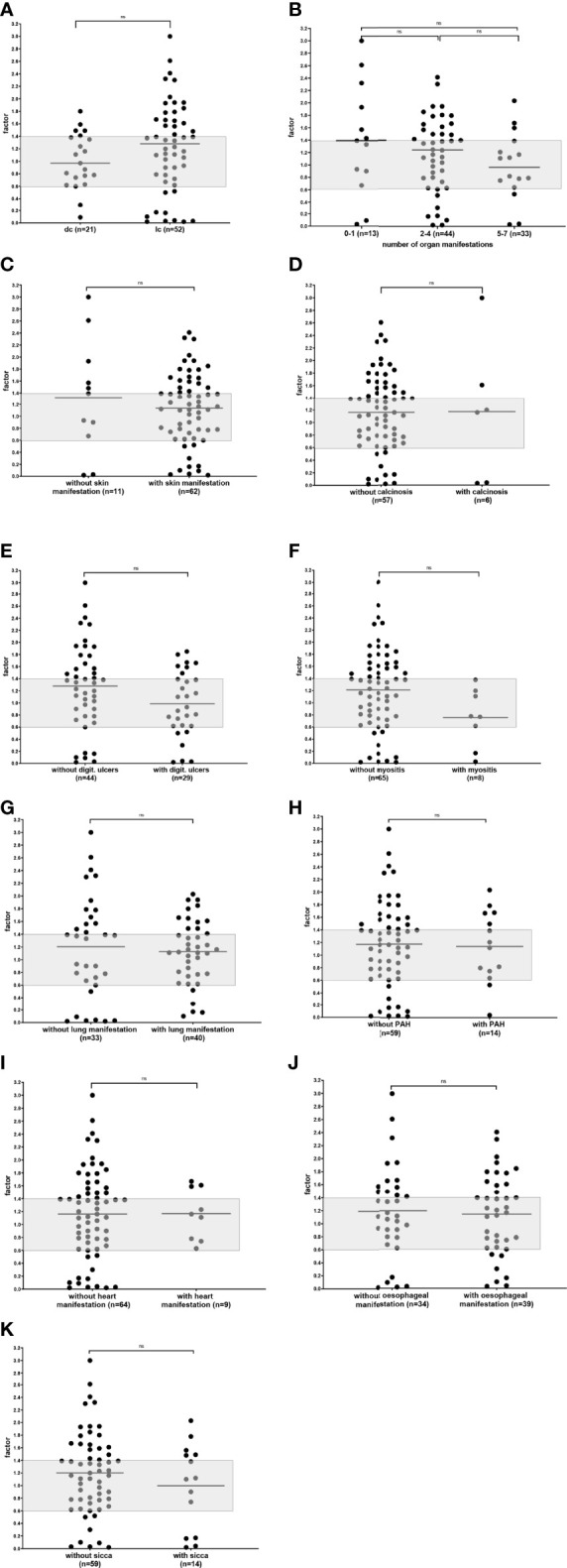
Reactivity of functional anti-AT_1_R antibodies in SSc patients comparing patients with lcSSc and dcSSc **(A)**, different numbers of organ manifestations **(B)**, patients with and without skin manifestations **(C)**, calcinosis **(D)**, digital ulcers **(E)**, myositis **(F)**, lung manifestations **(G)**, pulmonary arterial hypertension (PAH) **(H)**, heart manifestation **(I)** esophageal manifestation **(J)**, and sicca-syndrome **(K)**. Individual values are given reflecting a factor calculated with the mean of healthy individuals. The grey zone indicates the normal range; n.s., not significant; **-** median.

There was no significant difference in the prevalence or reactivity of inhibitory and stimulatory anti-AT_1_R antibodies comparing patients with dc and lcSSc (**Figure 2** and **Supplement Table S4**) The antibodies did also not correlate with the mRSS (r=-0.17, not significant; **Supplement Figure S3**).

Moreover, there were no significant differences in reactivity and prevalence of the antibodies comparing the number of organ manifestation, patients without and with skin manifestation, calcinosis, digital ulcers, myositis, lung manifestations, PAH, heart manifestation, esophageal manifestation, or sicca-syndrome (**Figure 2** and **Supplement Table S4**)

### Antibodies to AT_1_R, ET_A_1 and Topo-I Detected by ELISA

#### Prevalence and Reactivity in Patients With SSc and Other Disorders

Anti-AT_1_R antibodies detected by the commercially available ELISA kits were - like the functionally active antibodies – not confined to SSc and also found in patients with other collagen disorders and autoimmune and non-autoimmune liver diseases. Similar data were obtained for antibodies against the ET_A_1 (**Table 2**). Thus, there was a significant difference in reactivity of anti-AT_1_R-antibodies only between SSc and healthy controls. Patients with MCTD had even higher anti-AT_1_R antibodies than SSc-patients. In contrast, both, anti-AT_1_R and anti-ET_A_1-antibodies were hardly detected in PM and RA (**Figures 1B, C**).

**Table 2:** Prevalence of antibodies to topo-I, AT_1_R, ET_A_1 measured by ELISA in sera from patients with different disorders.

Diagnosis		Number tested	Antibodies to		
			AT_1_R	ET_A_1	Topo I
			Number (%) positive		
SSc	total	98	54 (55)	25 (26)	46 (47)
	Anti-Scl70 positive (ID)	31	25 (81)	11 (35)	22 (71)
	Antibodies to nucleoli (IFT	25	13 (52)	9 (36)	11 (44)
	Antibodies to centromeres (IFT)	31	9 (29)	2 (6)	7 (23)
	No antibodies	11	7 (64)	4 (36)	6 (55)
mixed connective tissue disease		27	18 (67)	10 (37)	0^***^
primary Sjoegren disease		54	22 (41)	12 (22)	1 (2)^***^
rheumatoid arthritis		54	6 (11)^****^	1 (2)^****^	0^***^
polymyalgia rheumatica		25	1 (4)^****^	0^**^	0^***^
PBC		30	9 (30)^*^	5 (17)	2 (7)^**^
PSC		31	13 (42)	3 (10)	0^***^
AIH		35	19 (52)	3 (9)	0^***^
alcoholic liver disease		20	9 (45)	2 (10)	0^***^
viral hepatitis		20	8 (40)	0^**^	0^***^
blood donors		15	0^***^	0^*^	0^***^

Significant as compared to patients with SSc: ^*^p < 0.05; ^**^p < 0.01; ^***^p < 0.001; ^****^p < 0.0001.

ID, immunodiffusion; ELISA, enzyme linked immunosorbent assay; IFT, immunofluorescence test.

Reactivity of both antibody types did also not significantly differ between SSc- patients and patients with autoimmune and non-autoimmune liver disorders (**Figures 1B, C**). The antibodies occurred in up to 50% of patients with autoimmune liver disorders and even in patients with toxic and viral liver diseases (up to 45%). In the competitive ELISA, anti-AT_1_R antibodies were also found in patients with other connective tissue disorders besides SSc as well as in autoimmune liver diseases (data not shown).

In contrast, anti-topo-I antibodies were highly specific for SSc and hardly found in patients with other disorders (**Table 2**).

Sensitivities of 55%, 28%, and 47%, and specificities of 66%, 87%, and 99% were calculated for the ELISAs measuring anti-AT_1_R-, -ET_A_1-, and anti-topo-I antibodies, respectively. ROC revealed for anti-AT_1_R-antibodies a significant difference to healthy controls for all diseases except for pSS (**Supplement Table S3**). Similar data were obtained for the anti-ET_A_1-antibodies while, again, anti-topo-I-antibodies were confined to SSc (not shown).

Within the group of SSc-patients, antibodies to topo-I were, as expected, preferentially associated with antibodies to Scl70 in the immunodiffusion (**Table 2**), but were also detected in sera showing antibodies to nucleoli or being antibody negative while they were hardly associated with ACA. A similar distribution was observed for anti-AT_1_R- and anti-ET_A_1 antibodies (**Table 2**).

#### Correlation Between Antibodies Against AT_1_R, ET_A_1, and Topo-I and Clinical Manifestations in SSc

**Figure 3:**
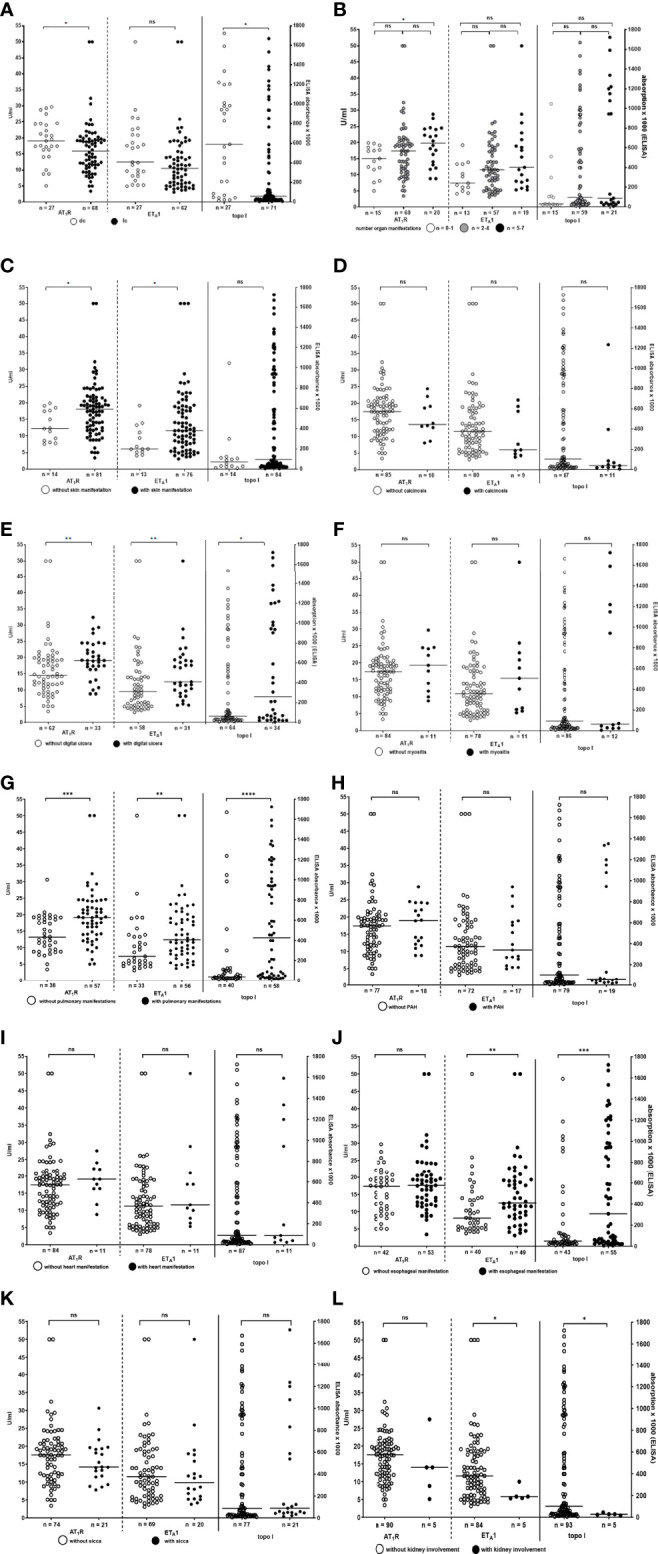
Reactivity of antibodies to AT_1_R, ET_A_1, and topo-I measured by ELISA in SSc patients comparing patients with lcSSc and dcSSc **(A)**, patients with different numbers of organ manifestations **(B)**, patients with and without skin manifestations **(C)**, calcinosis **(D)**, digital ulcers **(E)**, myositis **(F)**, lung manifestations **(G)**, pulmonary arterial hypertension (PAH) **(H)**, heart manifestation **(I)** esophageal manifestation **(J)**, and sicca-syndrome **(K)**. Individual values are given. Anti-topo-I abs refer to the left, anti-AT_1_R- and –ET_A_R-abs to the right y-axes. **­**- median. n.s., not significant; *p < 0.05; **p < 0.01; ***p < 0.001.

Patients with dcSSc showed significantly higher antibody reactivity and prevalence to topo-I and to vascular receptors than patients with lcSSc (**Figure 3A** and **Supplement Table S5**).

There was only a weak association of number of organs involved in SSc and reactivity of anti-AT_1_R-, –ET_A_1-, and -topo-I-antibodies (statistically significant for anti-AT_1_R and –topo-I antibodies, **Figure 3B** and **Supplement Table S5**).

Presence of skin manifestations slightly correlated with anti-AT_1_R- and -ET_A_1-antibody reactivity (**Figure 3C**). Calcinosis was not associated with a distinct antibody type while in patients with digital ulcers reactivity of all three antibody-types was significantly higher than in patients without (**Figures 3D, E** and **Supplement Table S5**). There was a weak correlation between the mRSS and anti-AT_1_R-(r=0.32; p<0.01), anti-ET_A_1- (r=0.34, p<0.01), and anti-topo-I-reactivity (r=0.47; p<0.001) (**Supplement Figure S3**).

In patients with pulmonary manifestations reactivity and prevalence of antibodies to AT_1_R, ET_A_1 and topo-I was significantly higher than in patients without pulmonary manifestations. However, there was no correlation of the antibody reactivity with the presence or absence of PAH (**Figures 3G, H** and **Supplement Table S5**).

Patients with esophageal manifestation had significantly higher antibody reactivity and prevalence to topo-I and ET_A_1 than patients without while there was no difference in reactivity towards AT_1_R (**Figure 3J** and **Supplement Table S5**)

Interestingly, patients with kidney involvement had significantly lower anti-topo-I- and - ET_A_1-antibody reactivity than patients without. This was not observed for antibodies to AT_1_R. However, it has to be considered that in only four patients the kidney was affected (**Figure 3L** and **Supplement Table S5**).

In patients with myositis, heart manifestations, and sicca-syndrome antibody prevalence and reactivity towards AT_1_R, ET_A_1 or topo-I did not differ from that in patients without these symptoms (**Figures 3F, I, K** and **Supplement Table S5**).

### Correlation Between Anti-AT_1_R Antibodies Detected by the Luminometric Assay and Anti- AT_1_R -, Anti-ET_A_1- and Anti-Topo-I Antibodies Detected by ELISA

**Figure 4:**
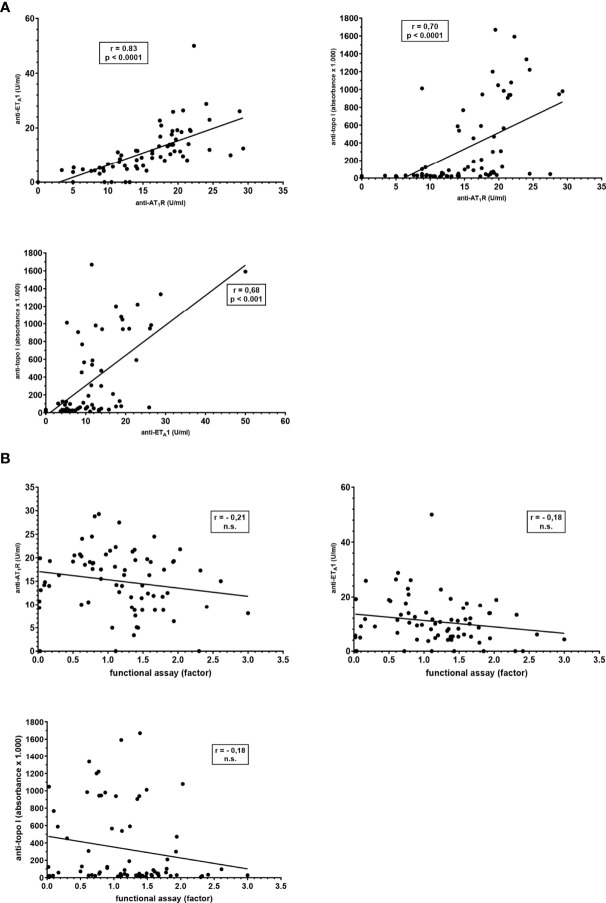
Correlation between reactivities towards AT_1_R, ET_A_1, and topo-I measured by different methods in sera from 73 patients with SSc. **(A)** Comparison of the antibody reactivities towards AT_1_R, ET_A_1, and topo-I measured by ELISA. **(B)** Comparison of the anti-AT1R reactivities determined by the functional luminometric assay and anti-AT_1_R-, -ET_A_1-, and -topo-I-antibodies determined by ELISA. Slopes, Spearman r correlations and significance levels are given.

Interestingly, there was a strong correlation between all three antibodies detected by ELISA, i.e. anti-topo-I-, anti-AT_1_R- and anti-ET_A_1-antibodies (anti-topo-I/anti-AT_1_R: r=0.70; anti-topo-I/anti-ET_A_1: r=0.68; anti-AT_1_R/anti-ET_A_1: r=0.83; for all p<0.001) (**Figure 4A**). In contrast, reactivity of the functionally active anti-AT_1_R-antibodies did not correlate with any of the antibodies determined by ELISA (anti-AT_1_R: r=-0.21, n.s.; anti- ET_A_1: r=-0.18, n.s.; anti-topo-I: r=-0.18, n.s.) (**Figure 4B**).

Anti-AT_1_R antibody reactivity measured by the competitive assay did also not correlate with that determined by any of the other assays (vs functional AT_1_R-assay: r=0.36, n.s.; vs AT_1_R-ELISA: r=0.33, n.s.; vs ET1-ELISA: r=0.09, n.s.; vs topo I-ELISA: r=-0.08, n.s.; data not shown).

### Purity of Plasma Membranes

Considering the high correlation between the three ELISAs measuring completely different autoantibodies we wanted to see whether purified plasma membranes overexpressing the AT_1_R may still contain further autoantigens. Therefore, the plasma membrane fractions from CHO-K1- and Huh7-cells were analyzed by Western blotting with sera from patients known to contain antibodies to other autoantigens such as the mitochondrial pyruvate dehydrogenase complex (PDC), topoisomerase-I or ribonucleoproteins. It became evident that the antigen fractions were still contaminated with cytoplasmic or nuclear antigens (**Supplement Figure S1**).

## Discussion

This study has three unique features: 1. It is the largest study analysing the relevance of functionally active, i.e. inhibitory or stimulatory antibodies to the AT_1_R in patients with SSc as well as other disorders. 2. It is the first study showing that anti-AT_1_R antibodies occur also in other collagen disorders than SSc and even in various liver disorders. 3. It compares and correlates directly the clinical relevance of antibodies to the AT_1_R, ET_A_1 and topoisomerase-I in SSc patients.

In the present study, the demonstration of functionally active antibodies was based on a luminometric method with the human cell line Huh7 constitutively expressing the AT_1_R. This assay had been established in a first step with CHO-cells overexpressing the receptor after transfection with an AT_1_R plasmid. Since results obtained with both cell lines were similar, we used in further experiments the Huh7 cells in order to remain in a human system. The assay proved to be reproducible and reliable with a intra- and interassay coefficient of variation of 20-25%. This is acceptable for a bioassay. Nevertheless one has to state that this assay is suitable for testing a large number of samples for research purposes but not for application in routine diagnostic.

In patients with SSc we found especially stimulatory antibodies (34% of the patients). However, neither the stimulatory nor the inhibitory antibodies were associated with any distinct clinical manifestations of SSc. Most importantly, they were detected also in other collagen disorders especialls MCTD and pSS. This is in line with the observation by Ilgen et al. (33) using a competitive ELISA for the demonstration of anti-AT_1_R antibodies, who also did not observe a correlation between the antibodies and clinical manifestations. Interestingly, we detected these functionally active antibodies also in another vascular disease, namely polymyalgia rheumatica while they were nearly absent in rheumatoid arthritis.

We, therefore, wanted to see whether functionally active anti-AT_1_R may occur also in another disease complex and selected patients with chronic liver disorders in which we had observed another functional antibody directed against the muscarinic receptor type 3 in a previous study (11, 34). Indeed, up to 50% of patients with autoimmune liver disorders and even up to 70% of patients with toxic or viral hepatitis had stimulatory or inhibitory anti-AT_1_R antibodies.

As already mentioned in the introduction, the first studies on anti-AT_1_R- and -ET_A_1 antibodies in patients with different disorders were based on true functional assays, namely bioassays that involved spontaneously beating cultured rat cardiomyocytes, or human endothelial cells (13–17). They have been replaced meanwhile by solid phase assays using extracts from CHO-cells overexpressing the human AT_1_R or ET_A_1 (15). In SSc, an association of antibodies detected by these assays and severe manifestations and complications of the disease, such as PAH, lung fibrosis, or digital ulcers has been reported (15, 21, 22, 33, 35), and they have been, therefore, claimed as ‘functional’ antibodies. However, they occur also in other disorders (14, 16–19, 24) and, as shown in the present study, also in other collagen disorders and chronic liver disorders. Antibodies reacting with those membrane-bound receptors in the ELISA – although correlating probably with clinical symptomes - are not necessarily functionally active because they may be directed against epitopes which are not involved in processes leading to inhibition or stimulation of the receptor. This may be an explanation why we did not observe a correlation between the reactivity of the functionally active anti-AT_1_R antibodies measured by luminescence assay and those detected by ELISA.

Although we did not observe an association of the functionally active antibodies with clinical manifestations, there was a correlation of anti-AT_1_R- and anti-ET_A_1- antibodies determined by ELISA with some manifestations especially pulmonary, esophageal or skin manifestations confirming previous studies. Also the reported association of anti-topo-I antibodies with disease severity, i.e. interstitial pneumonia, dcSSc, skin thickness severity, and skin sclerosis (2, 36–40), became evident in the present study.

The strong correlation between anti-topo-I-, anti-AT_1_R- and -ET_A_1 antibody reactivity measured by ELISA found in the present study is intriguing considering the fact that the antigens are completely different. A correlation between anti-AT_1_R- and -ET_A_1 antibodies has been reported already by Riemekasten et al. (15). It has been postulated that it is due to the natural ability of antibodies to bind to multiple antigens or a general B-cell hyperreactivity.The strong correlation of the antibodies to vascular receptors by ELISA with anti-topo-I antibodies shown in the present study suggests another explanation. Thus, one has to be aware that even very pure membrane preparations may be slightly contaminated with low amounts of nuclear or cytoplasmic antigens; indeed, we showed by Western blotting that our highly purified plasma membrane fractions from both, CHO- and Huh-7 cells still contained mitochondrial antigens as for instance the pyruvate-dehydrogenase complex, an important autoantigen in primary biliary cholangitis, as well as topoisomerase-I or ribonucleoproteins such as snRNP68; this explains the rather high prevalence of positive reactions obtained with these commercial assays with sera from patients with other autoimmune disorders being associated with autoantibodies directed against different nuclear or cytoplasmic antigens such as MCTD or autoimmune liver disorders; this hypothesis is underlined by the observation that patients with PM, a disease without any specific autoantibodies, or RA (associated with antibodies to extracellular matrix-associated antigens as for instance cyclic citrullinated protein) were nearly negative. I.e. the ‘anti-AT_1_R’ or ‘anti-ET_A_1’ antibodies detected by the commercial ELISA in different disorders may be directed actually against other antigens present in the plasma membrane fractions used to coat the ELISA plates and especially in SSc due to an contamination with topo-I.

Considering the fact that the functionally active anti-AT_1_R antibodies measured by the bioassay did not correlate with disease activity and manifestations of SSc and were found also in patients with other disorders, their pathogenic and clinical relevance for SSc remains, questionable. *In vitro* it has been shown that anti-AT_1_R antibodies induce ERK 1/2 phosphorylation and increase expression of transforming growth factor-beta (TGFß) messenger RNA expression, vascular cell adhesion molecule 1 and interleukin 8 in endothelial cells (12, 15, 21). These data fit to our observation that in SSc predominantly stimulatory antibodies were found. Moreover, it is known that neutrophils express the AT_1_R, and it has been, therefore, argued that the antibodies may activate neutrophil AT_1_R within blood vessels and that these activated neutrophils home to areas of inflammation and exacerbate tissue damage (25). However, this does not explain the occurrence of inhibitory antibodies which we also found in the SSc patients. It is still unknown whether these antibodies detected *in vitro* may interact with the receptor also *in vivo.*

There exists also not yet a conclusive explanation why anti-topo-I-antibodies correlate with some clinical SSc-symptomes. There are some studies indicating that they may influence fibroblast function in SSc (41–43). Interestingly, topotecan, an inhibitor of topoisomerase-I used in cancer therapy has been shown to induce SSc-like disease (44).

However, autoantibodies are not necessarily pathogenic. They may be even protective or belong to the pool of naturally occurring antibodies (45–47). This has, indeed, been postulated for antibodies to G-protein coupled receptors (GPCR). It has been argued that the secretion of anti-GPCR antibodies may suppress excessive immune responses and prevent tissue damage (48). The hypothesis that the functionally active anti-AT_1_R-antibodies may belong to the pool of naturally occurring antibodies is underlined by our observation that they have been been detected in a rather high prevalence of 70% in patients with viral and toxic liver diseases. It is well known that viral or bacterial infections increase the production of natural autoantibodies (49, 50), and this may also explain the occurrence of antibodies to vascular receptors in COVID-19 infection (26). Why those antibodies may become pathogenic under certain condition and how they are induced is still a matter of debate.

Our study has several limitations. Thus, number of patients with distinct clinical manifestations showing vascular antibodies were rather low, which may affect the statistical power. One has also to be aware that the determination of the prevalence of an antibody always depends upon the definition of cut off values. For the bioassay a factor of < 0.6 and > 1.6 for inhibitory and stimulatory antibodies, respectively was accepted which resulted in at most 15% of healthy controls showing either inhibitory or stimulatory antibodies. For the detection of antibodies to vascular receptors by ELISA we followed the instructions given by the manufacturers (cut off > 17) after verifying it in our settings with healthy controls; therefore, data are compatible with those reported in the literature. With respect to the functional anti-AT_1_R antibodies it is important to keep in mind that there exist inhibitory, stimulatory and neutral antibodies to the same receptor in one serum reacting with different epitopes (51). Therefore, in patients’ sera we can always detect only the predominating form of antibodies. Immunoglobulins from patients showing in our functional assay ‘no effect’ on the receptor may contain both, stimulatory and inhibitory antibodies in a similar concentration; i.e. we can also not exclude that healthy individuals contain functionally active anti-AT_1_R antibodies and that the stimulatory and inhibitory antibodies balance out one another (52). The predominance of one type of antibodies may then be indicative for an immunological dysbalance, but whether this is correlated with clinical symptomes remains still obscure.

In conclusion, we succeded to establish a reliable and reproducible assay for the demonstration of functionally active anti-AT_1_R antibodies. Although these antibodies might explain theoretically some of the symptoms of SSc, we did not find a correlation with clinical manifestations or symptoms. There was no correlation between the bioassay and an ELISA using AT_1_R-overexpressing membranes, indicating that the latter assay does not detect functionally active antibodies. Unexpectedly, we found that functional anti-AT_1_R-antibodies occur also in other collagen disorders and even liver disorders. This phenomenon has to be elucidated in further studies. Moreover, it became evident that anti-topo-I antibodies are superior to the anti-receptor antibodies especially with respect to disease specificity but also to correlation with clinical activity and organ manifestations.

## Data Availability Statement

The raw data supporting the conclusions of this article will be made available by the authors, without undue reservation.

## Ethics Statement

The studies involving human participants were reviewed and approved by Ethical committee of the University of Tuebingen (No. 076/212BO1; 647/2016BO2). Written informed consent to participate in this study was provided by the participants’ legal guardian/next of kin.

## Author Contributions

LB and BP established the assays, performed the experiments, acquired and analyzed data. NB performed the ELISAs. RK designed and coordinated the study. A-CP and JH provided patients’ sera and clinical data. BP, LB, A-CP, JH, and RK interpreted the data. RK and BP wrote the manuscript. All authors contributed to the article and approved the submitted version.

## Conflict of Interest

The authors declare that the research was conducted in the absence of any commercial or financial relationships that could be construed as a potential conflict of interest.

## Publisher’s Note

All claims expressed in this article are solely those of the authors and do not necessarily represent those of their affiliated organizations, or those of the publisher, the editors and the reviewers. Any product that may be evaluated in this article, or claim that may be made by its manufacturer, is not guaranteed or endorsed by the publisher.
