# Supplementary material for: Functionally Active Antibodies to the Angiotensin II Type 1-Receptor Measured by a Luminometric Bioassay Do Not Correlate With Clinical Manifestations in Systemic Sclerosis: A Comparison With Antibodies to Vascular Receptors and Topoisomerase I Detected by ELISA
Source: Front Immunol. 2021 Dec 9;12:786039. doi: 10.3389/fimmu.2021.786039 (PMC8695427; doi:10.3389/fimmu.2021.786039)
Supplement: Supplementary file 1 [file DataSheet_1.docx]

**Supplement**

**Clinical relevance of functional antibodies to the angiotensin II type1-receptor in systemic sclerosis detected by a new luminometric assay**

Lukas Bankamp, Beate Preuß, Ann-Christin Pecher, Nicola Beucke, Jörg Henes, Reinhild Klein

**Methods**

**Tables**

**Table S1 Clinical data in 98 patients with SSc**

| **Clinical parameters in 98 SSc patients** | | | | |  |
| --- | --- | --- | --- | --- | --- |
| Age at first manifes­ta­tion (years) | | | | mean+SD | 54 + 15 |
|  |  |  |  | median | 57 |
|  |  |  |  | range | 17 - 83 |
| Sex | | | | females: number (%) | 87 (89%) |
|  | | | | males: number (%) | 11 (11%) |
| Duration of disease at last visit (years) | | | | mean+SD | 10 + 7 |
|  |  |  |  | median | 7 |
|  |  |  |  | range | 0.5 - 28 |
| Kind of SSc | | | | lc: number | 71 |
|  | | | | dc: number | 27 |
| mRSS | | | | mean+SD | 7.7 + 7.3 |
|  | | | | median | 5 |
|  | | | | range | 0 - 30 |
| Organ manifestations (total number) | | | | mean+SD | 3.2 + 1.6 |
|  |  |  |  | median | 3 |
|  |  |  |  | range | 0 – 7^a)^ |
| Skin manifestations: number | | | | | 84 |
|  | | | Calcinosis: number | | 11 |
|  | | | Digital ulcers: number | | 34 |
| Lung involvement: number | | | | | 58 |
|  | Pulmonary arterial hypertension: number | | | | 19 |
| Esophagus: number | | | | | 55 |
| Muscles (myositis): number | | | | | 12 |
| Kidney: number | | | | | 4 |
| Heart: number | | | | | 11 |
| Exocrine glands (sicca syndrome): number | | | | | 21 |
| Antibodies to | | Scl70 (immunodiffusion): number | | | 31 |
|  | | Centromeres (ACA; IFT): number | | | 31 |
|  | | Nucleoli (fibrillarin; IFT): number | | | 25 |

^a)^ 4 patients had no organ manifestation and suffered only from Raynaud syndrome.

Five patients suffered additionally from primary biliary cholangitis as shown by the pr­e­sence of antimitochondrial antibodies of the anti-M2 type reacting with PDC-E2 and eleva­tion of cho­lestatic enzymes. In two patients, liver biopsy was performed revealing PBC stage I/II

**Table S2** Age and sex distribution in patients with different disorders analyzed for functionally active antibodies to the AT_1_R.

| Diagnosis | Number patients tested | Age (mean)  years | Age (range) years | Number fe­males/ males | Ratio f:m |
| --- | --- | --- | --- | --- | --- |
| SSc | 73 | 54,0 | 17-78 | 62/11 | 5,6 : 1 |
| mixed connective tissue disease (MCTD) | 25 | 38,6 | 10-69 | 18/7 | 2,6 : 1 |
| primary Sjoegren disease (pSS) | 24 | 49,8 | 18-84 | 23/1 | 23 : 1 |
| systemic lupus erythematosus (SLE) | 21 | 44,7 | 26-77 | 19/2 | 9,5 : 1 |
| rheumatoid arthritis (RA) | 24 | 61,0 | 33-80 | 18/6 | 6 : 1 |
| polymyalgia rheumatica (PM) | 16 | 65,6 | 43-80 | 8/8 | 1 : 1 |
| autoimmune liver diseases (PBC, PSC, AIH) | 97 | 47,9 | 17-78 | 67/30 | 2,2 : 1 |
| none autoimmune liver diseases (toxic, vi­ral) | 59 | 53,0 | 20-81 | 22/37 | 1 : 1,7 |
| blood donors | 36 | 31,2 | 20-69 | 18/18 | 1 : 1 |

**Table S3** ROC analysis for functionally active anti-AT1R-antibodies and anti-AT1R antibodies detected by ELISA: comparison of the different disorders with healthy controls

| Diagnosis | Number patients tested | Anti-AT1R antibodies detected by | | | |
| --- | --- | --- | --- | --- | --- |
|  |  | Functional lumino­metric assay | | ELISA | |
|  |  | AUC | p | AUC | p |
| SSc | 73 | 0.63 | **0.03** | 0.68 | **0.03** |
| mixed connective tissue disease (MCTD) | 25 | 0.68 | **0.02** | 0.93 | **0.0001** |
| Primary Sjoegren disease (pSS) | 24 | 0.51 | 0.88 | 0.66 | 0.08 |
| Systemic lupus erythematosus (SLE) | 21 | 0.55 | 0.49 | 0.86 | **0.0002** |
| Rheumatoid arthritis (RA) | 24 | 0.63 | 0.09 | 0.76 | **0.006** |
| Polymyalgia rheumatica (PM) | 16 | 0.68 | **0.04** | 0.79 | **0.006** |
| Autoimmune liver diseases (PBC, PSC, AIH) | 97 | 0.51 | 0.83 | 0.69 | **0.02** |
| None autoimmune liver diseases (toxic, vi­ral) | 57 | 0.56 | 0.37 | 0.77 | **0.002** |

AUC: area under the curve

**Table S4** Correlation between functional anti-AT_1_R antibodies and clinical symptoms in 73 pa­tients with SSc

| Clinical symptoms | | number tested | Antibodies to AT_1_R | | |
| --- | --- | --- | --- | --- | --- |
|  |  |  | total | inhi­b­i­tory | stimula­tory |
|  |  |  | Number (%) positive | | |
| dc |  | 21 | 8 (38) | 3 (14) | 5 (24) |
| lc |  | 52 | 30 (57) | 10 (19) | 20 (38) |
| Number organ manifestations | 0-1 | 13 | 8 (62) | 2 (17) | 6 (46) |
|  | 2-4 | 44 | 24 (54) | 8 (18) | 16 (36) |
|  | 5-7 | 16 | 6 (38) | 3 (19) | 3 (19) |
| Skin manifestions | without | 11 | 7 (43) | 2 (18) | 5 (45) |
|  | with | 62 | 31 (31) | 11 (18) | 20 (32) |
| calcinosis | without | 57 | 34 (59) | 11 (19) | 23 (40) |
|  | with | 6 | 4 (66) | 2 (33) | 2 (33) |
| Digital ulcers | without | 44 | 23 (52) | 6 (14) | 17 (39) |
|  | with | 29 | 14 (48) | 6 (21) | 8 (28) |
| Lung manifestations | without | 33 | 20 (60) | 7 (21) | 13 (39) |
|  | with | 40 | 17 (43) | 5 (13) | 12 (30) |
| PAH | without | 59 | 30 (50) | 10 (17) | 20 (34) |
|  | with | 14 | 7 (50) | 2 (14) | 5 (36) |
| Heart manifestation | without | 64 | 34 (53) | 12 (19) | 22 (34) |
|  | with | 9 | 3 (33) | 0 | 3 (33) |
| Sicca syndrome | without | 59 | 28 (48) | 8 (14) | 20 (34) |
|  | with | 14 | 9 (64) | 4 (29) | 5 (36) |
| myositis | without | 65 | 35 (54) | 10 (15) | 25 (38) |
|  | with | 8 | 2 (25) | 2 (25) | 0 ^*^ |
| Esophageal manifestation | without | 44 | 23 (52) | 6 (14) | 17 (39) |
|  | with | 29 | 14 (49) | 6 (21) | 8 (28) |

* significant as compared to without myositis (p < 0.05)

**Table S5** Correlation between antibodies to AT_1_R, ET-1 and topo-I measured by ELISA and clinical symp­toms in 73 patients with SSc

| Organ involvement/symptoms | Number patients tested (AT_1_R-ET_A_1-Topo-I) | antibodies to | | | |
| --- | --- | --- | --- | --- | --- |
|  |  | AT_1_R | ET_A_1 | Topo-I | |
|  |  | Number (%) positive) | | | |
| SSc type | lc (n=68-62-71) | 33 (49) | 14 (23) | | 20 (28) |
|  | dc (n=27-27-27) | 19 (70) | 12 (44) | | 17 (63) |
| Number organ manifestations | 0-1(n=15-13-15) | 7 (47) | 0 | | 2 (13) |
|  | 2-4 (n=60-57-59) | 31 (52) | 17 (30) | | 26 (44) |
|  | 5-7 (n=20-19-21) | 13 (65) | 8 (42) | | 9 (43) |
| skin | without (n=14-13-14) | 5 (36) | 1 (8) | | 1 (7) **^*^** |
|  | with (n=81-76-84) | 46 (57) | 26 (34) | | 36 (43) |
| calcinosis | without (n=85-80-87) | 44 (55) | 23 (29) | | 35 (40) |
|  | with (n=10-9-11) | 4 (40) | 3 (33) | | 2 (18) |
| Digital ulcers | without (n=62-58-64) | 27 (44) | 12 (21) | | 20 (31) |
|  | with (n=33-31-34) | 25 (76) | 14 (45) | | 17 (50) |
| Lung | without (n=38-33-40) | 15 (39) | 5 (15) | | 5 (13) **^****^** |
|  | With (n=57-56-58) | 30 (53) | 21 (38) | | 32 (55) |
| Pulmonary arterial | without (n=77-71-79) | 42 (55) | 15 (21) | | 31 (39) **^**^** |
| hypertension | with (n=18-18-19) | 10 (56) | 6 (33) | | 6 (32) |
| esophageal | without (n=42-40-43) | 22 (52) | 6 (15) | | 9 (21) |
| manifestation | with (n=53-49-55) | 30 (57) | 19 (39) | | 28 (51) |
| myositis | without (n=84-78-86) | 45 (54) | 21 (27) | | 32 (37) |
|  | with (n=11-11-12) | 7 (64) | 5 (45) | | 5 (42) |
| Sicca syndrome | without (n=74-69-77) | 43 (59) | 21 (30) | | 30 (39) |
|  | with (n=21-20-21) | 9 (43) | 5 (25) | | 7 (33) |
| heart | without (n=84-78-87) | 44 (52) | 21 (27) | | 32 (38) |
|  | with (n=11-11-11) | 7 (64) | 5 (45) | | 5 (45) |
| kidney | without (n=90-84-93) | 49 (54) | 27 (32) | | 37 (40) |
|  | with (n=5-5-5) | 1 (20) | 0 | | 0 |
| Primary biliary cho­langitis | without (n=88-82-91) | 48 (55) | 25 (30) | | 37 (41) |
|  | with (n=7-7-7) | 3 (43) | 1 (14) | | 0 |

Significance levels between lc and dc as well as without and with the different organ mani­fe­stations: ^*^ =p<0.05; ^**^ = p< 0.01; ^***^ = p < 0.001; ^****^ = p < 0.0001

**Figure S1**

**
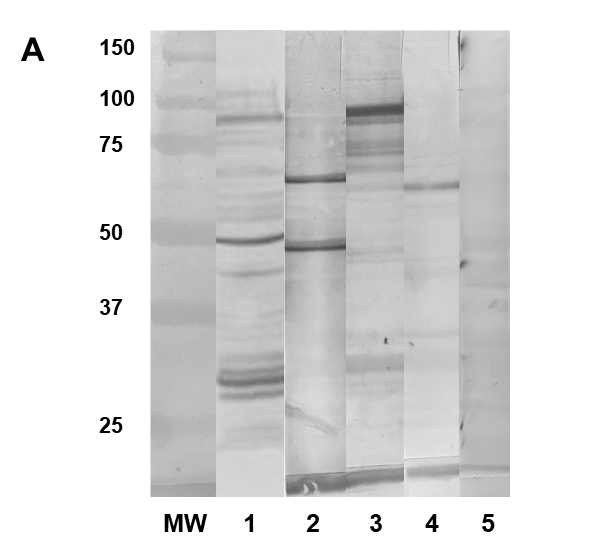
**

**25**

**37**

**50**

**250**

**75**

**150**

**250**

**
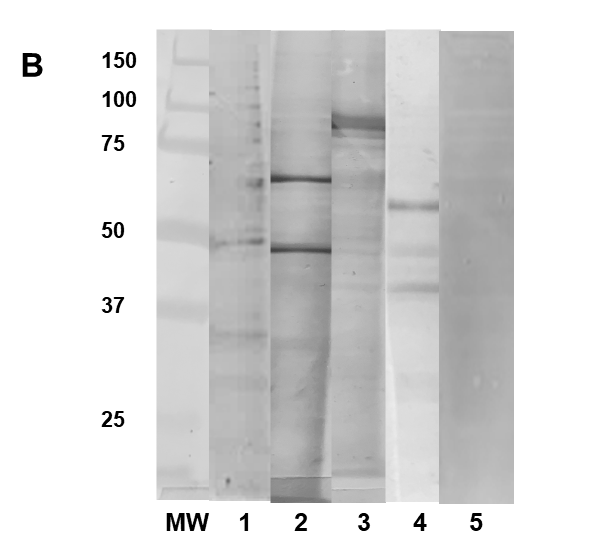
**

**Figure S1** Analysis of purified plasma membranes from CHO-cells (A) and Huh7-cells (B) for the expression of the angiotensin II receptor 1 and a contamination with non-membranous antigens by Western blotting

MW: molecular marker (Precision Plus Protein Dual Xtra Standards; BioRad, Hercules, CA, USA)

lanes 1-5: plasma membranes from CHO- (A) and Huh7 cells (B) tested against a anti-AT1R ab from rabbit (Biozol) reacting with the AT_1_R at about 47 kDa (1), a serum from a patient with primary biliary cholangitis with antimitochondrial antibodies directed to two antigens at 70 and 45 kDa of the pyruvate dehydrogenase complex (2), a serum from a patient with sys­temic sclerosis with anti-Scl70 antibodies reacting with topoisomerase I at 100 kDa (3), a se­rum from a patient with mixed connective tissue disease reacting with reacting with snRNP68 (4) and a normal serum from a healthy donor (5).

**Figure S2**

 **Figure S2** Inhibition of the AT_1_R expressed by CHO-cells transfected with this receptor and Huh7-cells expressing this receptor constitutively by the specific AT_1_R-antagonist losartan in different concentrations measured in the luminometric assay. Results are given as percent of untreated cells. Mean and standard deviation of fourfold determination are given.

**Figure S3**

**Figure S3**: Correlation between the mRSS and reactivity of functionally active antibodies to the AT1R (a), and of antibodies to AT_1_R (b), ET_A_1 (c) and topo I (d) measured by ELISA
